# Histone Deacetylase 3‐Progesterone Receptor Cooperation Regulates Progesterone Responsiveness in the Uterus

**DOI:** 10.1002/mco2.71122

**Published:** 2026-11-05

**Authors:** Sohyeon Yun, Rong Li, Soo Lim Kim, Loan Thi Kim Nguyen, Shamsun Nahar, Keun Cheon Kim, DaHye Kang, Eunhee M. Jeong, Lisa Brennaman, Mistie P. Mills, Steven L. Young, Bruce A. Lessey, Tae Hoon Kim, Jung‐Yoon Yoo, Jae‐Wook Jeong

**Affiliations:** ^1^ Department of Obstetrics, Gynecology and Women's Health University of Missouri Columbia Missouri USA; ^2^ Center For Reproductive Medicine and Obstetrics and Gynecology, Nanjing Drum Tower Hospital, Affiliated Hospital of Medical School Nanjing University Nanjing China; ^3^ Department of Obstetrics and Gynecology Duke University Durham North Carolina USA; ^4^ Department of Obstetrics and Gynecology Wake Forest Baptist Health Winston‐Salem North Carolina USA; ^5^ Department of Biomedical Laboratory Science Yonsei University Mirae Campus Wonju Republic of Korea

**Keywords:** endometriosis, histone deacetylase 3, progesterone receptor, progesterone resistance, uterine receptivity

## Abstract

Endometriosis affects a substantial proportion of women of reproductive age, with 30%–50% affected by infertility. A defining pathological feature is progesterone resistance resulting from impaired progesterone receptor (PGR) signaling, yet the underlying mechanisms remain incompletely understood. Here, we define conserved shared target genes of histone deacetylase 3 (HDAC3) and PGR in the mouse uterus using integrated transcriptomic and cistromic analyses, supported by uterine‐specific *Hdac3* and *Pgr* knockout mice. Co‐immunoprecipitation and proximity ligation assays indicate that HDAC3 associates with PGR, with reciprocal attenuation of their expression in *Hdac3*‐ and *Pgr*‐deficient uteri. Multiplex immunofluorescence demonstrates colocalization of HDAC3 and PGR in endometrial epithelial and stromal cells. HDAC3 and PGR expression levels show a positive correlation in stromal cells in both controls and infertile women with endometriosis, whereas this correlation in epithelial cells is selectively preserved in a subset of infertile cases. Chromatin profiling demonstrates extensive co‐occupancy of HDAC3 and PGR at regulatory regions associated with progesterone‐responsive genes. Together, these findings support a model in which HDAC3 functions as a coregulator of PGR‐dependent transcription in the uterus and indicate that HDAC3‐PGR cooperation is a key determinant of endometrial receptivity. This work provides mechanistic insight into progesterone resistance underlying endometriosis‐associated infertility.

## Graphical abstract

HDAC3 and PGR exhibit coordinated expression and chromatin occupancy in the receptive endometrium. Loss of HDAC3‐PGR cooperation in endometriosis disrupts progesterone‐responsive gene regulation and impairs uterine receptivity.

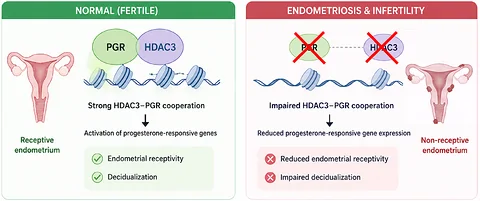

## 1 Introduction

Successful pregnancy requires the establishment of a receptive endometrium capable of supporting embryo implantation and decidualization, and impairment of these uterine functions represents a major cause of infertility [1]. Endometriosis is a chronic inflammatory disease characterized by ectopic growth of endometrial‐like tissue, affecting more than 10% of reproductive‐aged women and frequently associated with pelvic pain and infertility [2]. Endometrial receptivity is governed by coordinated steroid hormone receptor signaling and downstream transcriptional programs. Among these, progesterone receptor (PGR) plays a central role by opposing estrogen‐driven proliferation and coordinating with estrogen receptor α (ESR1), glucocorticoid receptor (GR), and other nuclear receptors [3, 4, 5]. Impaired PGR signaling and disrupted nuclear receptor crosstalk have been implicated in the pathogenesis of endometriosis, contributing to progesterone (P4) resistance and uterine dysfunction [3, 4].

PGR transcriptional activity is tightly regulated by epigenetic mechanisms and coregulatory complexes, including steroid receptor coactivators (SRCs), nuclear receptor corepressors (NCoR/SMRT), histone acetyltransferases (e.g., p300/CBP), and histone deacetylases (HDACs) [6, 7, 8]. Dysregulation of these nuclear receptor‐coregulator networks has been linked to impaired P4 responsiveness and endometrial dysfunction in endometriosis [9]. Among these epigenetic regulators, HDAC3 has emerged as a critical regulator of uterine receptivity. Uterine‐specific loss of HDAC3 results in epithelial hyperproliferation, decreased PGR protein expression, and a non‐receptive endometrium, directly linking HDAC3 dysregulation to P4 resistance and infertility [8].

P4 resistance is defined as the failure of endometrial tissue to respond appropriately to P4 stimulation despite adequate hormone availability and is a hallmark of endometriosis, occurring in both eutopic endometrium and ectopic lesions [10, 11]. Loss of P4 responsiveness disrupts the balance between estrogen‐driven proliferation and P4‐mediated differentiation, promoting lesion growth and impairing endometrial receptivity [1, 12, 13]. Although epigenetic modifications are increasingly recognized as key regulators of endometrial function and steroid hormone responsiveness [14], the molecular mechanisms that integrate epigenetic control with PGR‐dependent transcription remain poorly defined.

HDACs regulate chromatin accessibility through deacetylation of histone and non‐histone substrates, thereby modulating transcriptional activity [15, 16]. Within the class I HDAC family, HDAC1 and HDAC2 have been reported to regulate steroid hormones in endometriotic stromal cells [17]. HDAC3 is unique in its subcellular distribution across the nucleus, cytoplasm, and plasma membrane and in its ability to form transcriptional corepressor complexes with NCoR and SMRT to regulate a broad range of transcription factors, including thyroid and estrogen receptors [18, 19, 20]. Despite its established role in uterine biology, whether HDAC3 directly regulates PGR function remains unknown. Our previous work demonstrated that loss of uterine HDAC3 results in infertility due to impaired endometrial receptivity and decidualization [8]. We further observed that HDAC3 deficiency enhances epithelial proliferation and reduces PGR protein levels, suggesting a functional link between HDAC3 attenuation and P4 resistance. However, the mechanistic relationship between HDAC3 and PGR in the uterus remains undefined.

In this study, we provide evidence that HDAC3 associates with PGR and that their expression levels are positively correlated in the eutopic endometrium of both control women and infertile women with endometriosis, as well as in mouse models. Integrated transcriptomic and cistromic analyses reveal that HDAC3 and PGR co‐occupy conserved chromatin regions and coordinate the regulation of gene expression programs essential for uterine receptivity. Together, these findings identify HDAC3 as a critical coregulator of PGR‐dependent transcription and support a model in which disruption of HDAC3‐PGR cooperation underlies P4 resistance and endometriosis‐associated infertility.

## 2 Results

### 2.1 Downregulation of Stromal HDAC3 Correlates With Reduced PGR Expression in the Eutopic Endometrium From Infertile Women With Endometriosis

Our previous work demonstrated that HDAC3 expression is reduced in the eutopic endometrium from infertile women with endometriosis and that stromal HDAC3 is required to maintain endometrial homeostasis and receptivity [8]. Because P4 resistance is a defining pathophysiological feature of endometriosis [2], we examined the relationship between HDAC3 and PGR in eutopic endometrium collected during mid‐secretory phase from fertile women without endometriosis (*n* = 22) and infertile women with endometriosis (*n* = 43). Immunohistochemical analysis revealed a marked reduction in both PGR and HDAC3 protein levels in the stromal compartment of infertile women with endometriosis compared with controls (Figure 1A). Quantitative H‐score analysis demonstrated that stromal PGR levels were significantly decreased in infertile women with endometriosis (75.62 ± 8.05) compared to controls (156.40 ± 9.16, *p* < 0.001) (Figure 1B). Similarly, stromal HDAC3 expression was significantly reduced in these patients (119.2 ± 8.02) compared to controls (189.40 ± 10.35, *p* < 0.001). Notably, stromal HDAC3 and PGR expression levels exhibited a strong positive correlation (*r* = 0.6026, *p* < 0.001), indicating coordinated regulation within the stromal compartment (Figure 1B).

**FIGURE 1:**
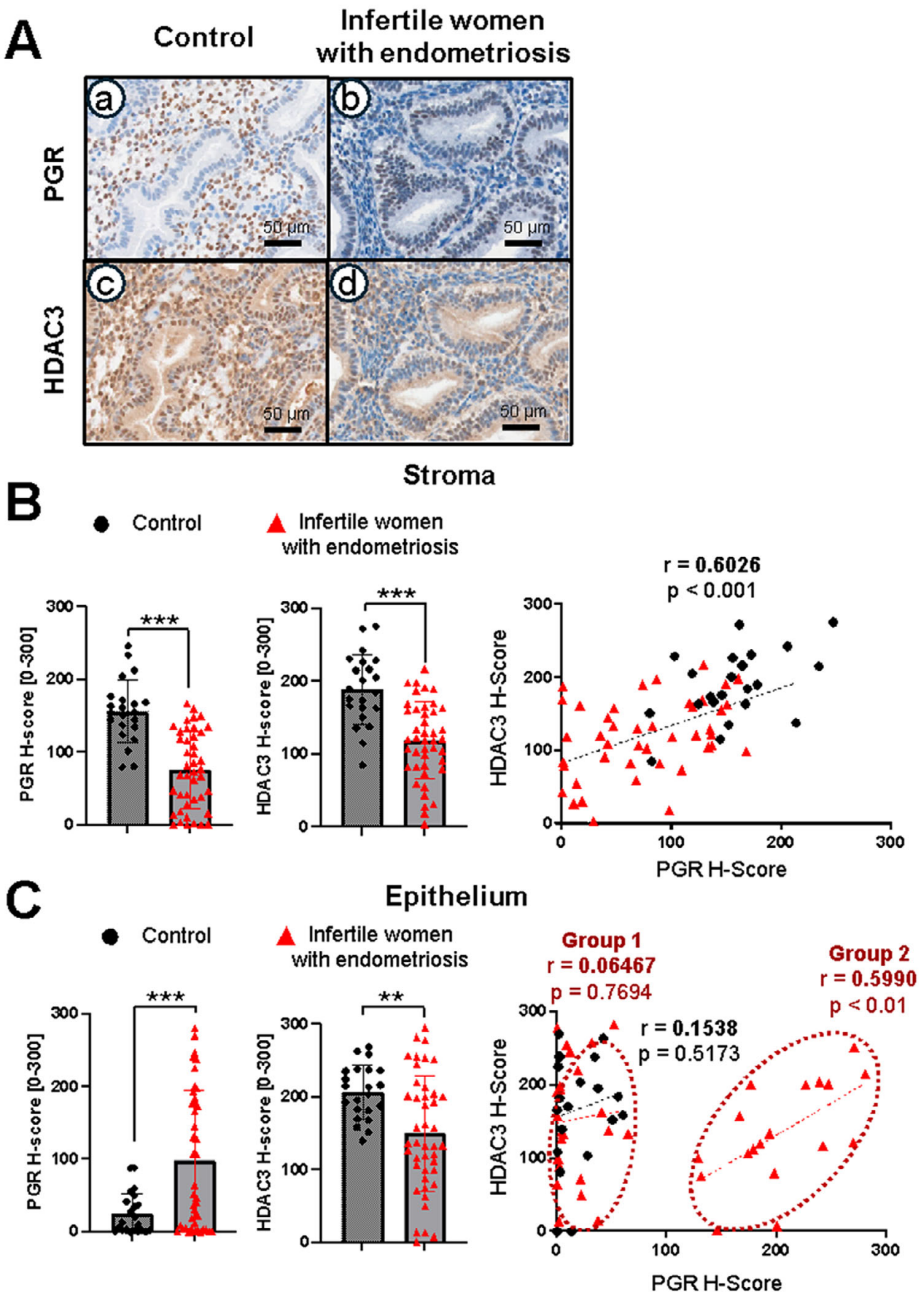
Coordinated reduction of PGR and HDAC3 protein expression in human endometrial stroma in infertile women with endometriosis during the mid‐secretory phase. (A) Representative images of immunohistochemistry (IHC) staining for PGR and HDAC3 from fertile women without endometriosis (controls, *n* = 22) and infertile women with endometriosis (*n* = 43). (B) Semi‐quantitative analysis of stromal PGR (left) and HDAC3 (middle) expression levels, presented as H‐score with a correlation plot of HDAC3 and PGR expression (right) in serial sections of human endometrium. (C) Semi‐quantitative analysis of epithelial PGR (left) and HDAC3 (middle) expression levels, presented as H‐scores, with correlation plot of HDAC3 and PGR expression (right) in the serial sections. Two epithelial subgroups were identified among infertile women with endometriosis: group 1 (low PGR) and group 2 (high PGR). Data are represented as mean ± SEM. * *p* < 0.05 and *** *p* < 0.001 by Student's *t*‐test. Correlations were assessed using Pearson's correlation coefficient (*r*).

In contrast, the HDAC3‐PGR relationship differed in the epithelial compartment. Epithelial HDAC3 expression was significantly decreased in infertile women with endometriosis (150.50 ± 12.10, *p* = 0.0024) compared to controls (207.00 ± 7.85) (Figure 1C). Notably, epithelial PGR expression exhibited heterogeneity within the endometriosis group. Group 1 (*n* = 23) exhibited low epithelial PGR expression (16.77 ± 4.06), comparable to controls (18.62 ± 4.62, *p* = 0.7658), and showed no significant correlation with HDAC3 (*r* = 0.06467, *p* = 0.7694). In contrast, Group 2 (*n* = 18) displayed significantly elevated epithelial PGR expression (202.60 ± 11.17, *p* < 0.001) and exhibited a significant positive correlation between epithelial HDAC3 and PGR (*r* = 0.5990, *p* < 0.01). Together, these data demonstrate coordinated downregulation of stromal HDAC3 and PGR in the eutopic endometrium of infertile women with endometriosis, together with heterogeneous epithelial PGR expression and distinct epithelial HDAC3‐PGR correlation patterns. These findings highlight compartment‐specific dysregulation of HDAC3‐PGR signaling associated with impaired P4 responsiveness.

### 2.2 HDAC3 and PGR are Coexpressed in Stromal and Epithelial Cells, With Compartment‐Specific Correlation Patterns Altered in Infertile Women With Endometriosis

Multiplex immunofluorescence enables the simultaneous detection of multiple proteins within the same tissue section, providing spatial and cell‐type‐specific resolution that cannot be achieved using serial IHC alone [21]. We performed multiplex immunofluorescence to directly assess HDAC3 and PGR coexpression within individual cells in eutopic endometrial tissues from fertile controls and infertile women with endometriosis (*n* = 3 per group). Endometriosis samples from epithelial PGR‐high cases (Group 2) were selected to enable cell‐level analysis of aberrant epithelial PGR regulation. Within each section, epithelial cells were identified by pan‐keratin staining (yellow), stromal fibroblasts by CD10 (cyan), PGR by green fluorescence, and HDAC3 by red fluorescence (Figure 2A). Consistent with previous reports [8, 22], control endometrium exhibited strong stromal PGR expression with relatively low epithelial PGR levels, along with strong HDAC3 expression in both stromal and epithelial compartments (Figure 2A(a–e)). In contrast, eutopic endometrium from infertile women with endometriosis displayed markedly increased epithelial PGR expression (Figure 2A(f–j)) and reduced stromal HDAC3 expression (Figure 2A(j)). Single‐cell quantitative analysis revealed a strong and consistent correlation between HDAC3 and PGR expression in stromal cells from both controls (*r* = 0.6787, 0.6736, and 0.7570; all *p* < 0.001) and infertile women with endometriosis (*r* = 0.7655, 0.6985, and 0.8506; all *p* < 0.001) (Figure 2B). In contrast, epithelial cells from control endometrium exhibited weak correlations between HDAC3 and PGR expression (*r* = −0.0961, 0.5046, and 0.4077; all *p* < 0.001), whereas epithelial cells from infertile women with endometriosis showed markedly strengthened correlations (*r* = 0.7149, 0.7471, and 0.8408; all *p* < 0.001) (Figure 2B). These findings reveal that HDAC3 and PGR expression are coexpressed at the single‐cell level in both stromal and epithelial compartments of the eutopic endometrium, with altered correlation patterns in infertile women with endometriosis. These compartment‐specific differences suggest that HDAC3‐PGR coordination is differentially regulated across cell types and disrupted in endometriosis, consistent with P4 signaling dynamics.

**FIGURE 2:**
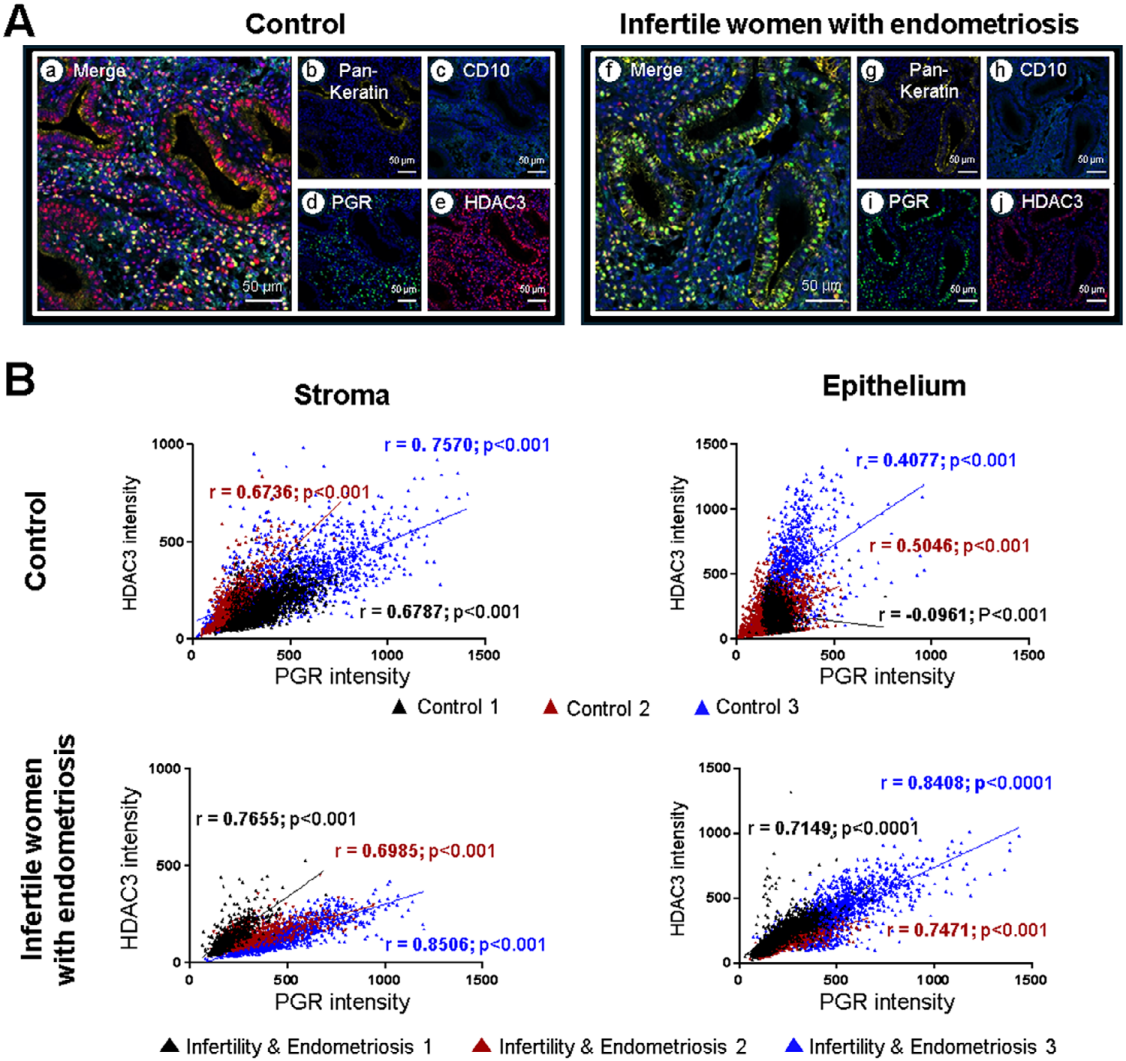
PGR and HDAC3 are coexpressed and exhibit correlated expression patterns in endometrial stromal and epithelial cells in infertile women with endometriosis during the mid‐secretory phase. (A) Representative images of multiplex immunofluorescence analysis of eutopic endometrial sections from fertile controls and infertile women with endometriosis (*n* = 3 per group). Yellow: Pan‐keratin; Cyan: CD10; green: PGR; red: HDAC3; blue: DAPI. (B) Quantitative correlation analysis between PGR and HDAC3 expression within epithelial and stromal compartments from control and endometriosis samples (*n* = 3). Correlations were assessed using Pearson's correlation (*r*) and corresponding *p*‐values shown for each sample.

### 2.3 Loss of Uterine HDAC3 Reprograms Transcriptional Networks Associated With Inflammation and Fibrosis

We previously generated a uterine‐specific *Hdac3* knockout (*Hdac3^d/d^*) mouse that exhibits infertility due to a non‐receptive endometrium [8]. To investigate the transcriptional consequences of HDAC3 loss and its potential impact on PGR‐dependent uterine function, we performed RNA‐Seq on uteri from *Hdac3^f/f^* and *Hdac3^d/d^* mice at gestation day (GD) 3.5, a critical window for uterine receptivity. Unsupervised hierarchical clustering and principal component analysis (PCA) demonstrated clear segregation of uterine transcriptomes between *Hdac3^f/f^* and *Hdac3^d/d^* mice (Figure 3A,B), indicating widespread transcriptional reprogramming following HDAC3 deletion. Pathway enrichment analysis revealed that loss of HDAC3 was associated with robust activation of immune‐related signaling pathways, including cytokine storm, S100 family signaling, macrophage alternative and classical activation signaling, and neuroinflammation signaling (Figure 3C). In parallel, multiple fibrosis‐associated pathways, including hepatic fibrosis, wound healing, and sclerosis signaling, were significantly upregulated. Additional enriched pathways included cAMP response element‐binding protein (CREB) signaling, liver X receptor/retinoid X receptor (LXR/RXR) activation, and G protein‐coupled receptor signaling. In contrast, kinetochore metaphase signaling pathway was downregulated (Figure 3C). Upstream regulator analysis further supported a shift toward a proinflammatory transcriptional state, predicting inhibition of β‐estradiol signaling and activation of multiple inflammatory mediators, including lipopolysaccharide, interferon gamma (IFNG), interleukin‐1β (IL‐1B), interleukin 6 (IL6), and tumor necrosis factor (TNF), accompanied by inhibition of signal transducer and activator of transcription 3 (STAT3) signaling (Figure 3D).

**FIGURE 3:**
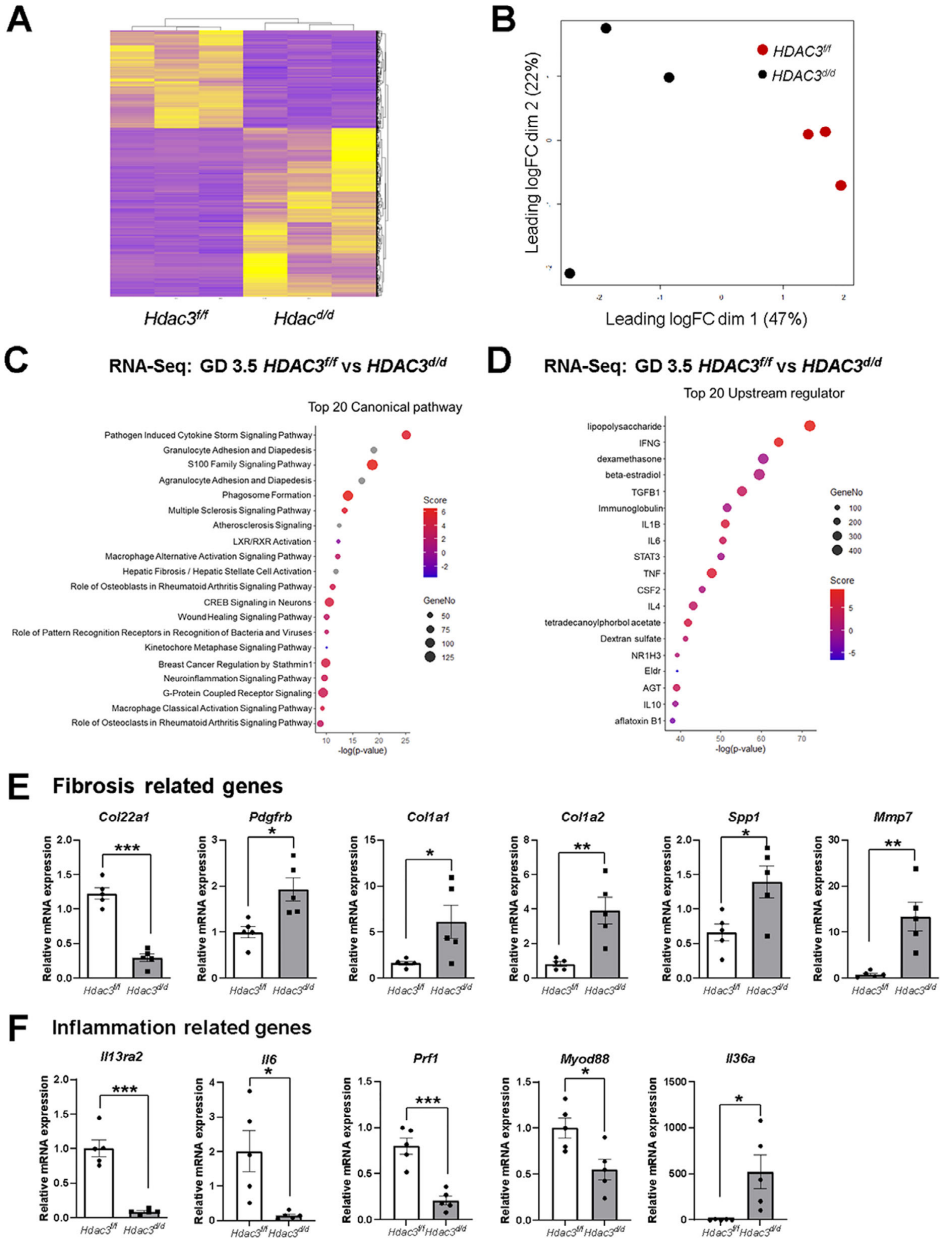
Transcriptomic analysis of *Hdac3^f/f^* and *Hdac3^d/d^* mouse uteri at GD3.5. (A) Heatmap demonstrating differentially expressed genes (DEGs) between *Hdac3^f/f^* and *Hdac3^d/d^* mouse uteri at GD3.5 (*n* = 3). (B) Principal component analysis (PCA) plot showing clear separation between the transcriptomic samples from GD3.5 *Hdac3^f/f^* and *Hdac3^d/d^* mouse uteri. (C) Dot plot depicting the top 20 canonical pathways enriched among the DEGs from GD3.5 *Hdac3^f/f^* and *Hdac3^d/d^* mouse uteri. (D) Dot plot showing the top 20 predicted upstream regulators of the DEGs. Pathway enrichment and upstream regulator analyses were performed using Ingenuity Pathway Analysis (IPA). Dot color indicates the z‐score, and dot size reflects the number of genes associated with each pathway. (E and F) RT‐qPCR validation of fibrosis (E) and inflammation (F) related genes in GD3.5 *Hdac3^f/f^* and *Hdac3^d/d^* mouse uteri. Data are presented as mean ± SEM. * *p* < 0.05, ** *p* < 0.01, *** *p* < 0.001 by Student's *t*‐test.

Based on the pathway enrichment analysis, we next validated the expression of selected fibrosis‐ and inflammation‐associated genes in uteri from *Hdac3^f/f^* and *Hdac3^d/d^* mice at GD3.5 using quantitative real‐time PCR. Fibrosis‐related genes, including *Collagen Type XXII Alpha 1* (*Col22a1*), *Platelet Derived Growth Factor Receptor Beta* (*Pdgfrb*), *Collagen Type I Alpha 1* (*Col1a1*), *Collagen Type I Alpha 2* (*Col1a2*), *Secreted Phosphoprotein 1* (*Spp1*), and *Matrix metalloproteinase 7* (*Mmp7*), were significantly upregulated in *Hdac3^d/d^* uteri compared to *Hdac3^f/f^* (Figure 3E). In parallel, expression of inflammation‐associated genes, including *Interleukin‐13 receptor alpha 2* (*Il13ra2*), *Il6*, *Perforin 1* (*Prf1*), *Myeloid differentiation primary response 88* (*Myd88*), and *Interleukin 36* (*Il36a*), was also significantly increased following HDAC3 deletion (Figure 3F). Given that P4 signaling suppresses inflammation [23], these findings suggest that enhanced immune and fibrotic gene expression following HDAC3 loss is associated with reduced PGR activity and impaired P4 responsiveness. Notably, inhibition of P4 signaling was among the top predicted upstream regulatory events, further supporting disrupted P4 responsiveness.

### 2.4 HDAC3 ChIP‐Seq Identifies Direct Chromatin Targets Associated With Inflammatory, Fibrotic, and P4 Signaling Pathways

HDAC3 is a histone deacetylase that binds to enhancer regions to regulate gene transcription [24]. To define the chromatin‐binding landscape of HDAC3 in the receptive uterus, we performed HDAC3 Chromatin Immunoprecipitation (ChIP)‐seq using uteri from *Hdac3^f/f^* mice at GD3.5. High‐confidence HDAC3 binding sites were identified as conserved peaks across replicates. Genes associated with these HDAC3 binding peaks were predicted using Genomic Regions Enrichment of Annotations Tool (GREAT) [25] and compared with the differentially expressed genes (DEGs) identified from the *Hdac3^d/d^* uterine RNA‐seq dataset (Figure 4). Notably, 46% (1306 of 2842) of DEGs induced by uterine HDAC3 loss were associated with at least one HDAC3 binding peak, indicating that a substantial fraction of transcriptional changes following HDAC3 deletion are potentially directly regulated at the chromatin level (Figure 4A). Pathway enrichment analysis of these putative HDAC3 target genes revealed significant overrepresentation of inflammatory and fibrotic signaling pathways, as well as β‐estradiol, STAT3, CREB, and LXR/RXR signaling pathways (Figure 4B,C). Importantly, inhibition of P4 signaling emerged as one of the top predicted upstream regulatory events among HDAC3‐bound DEGs (Figure 4C). These results indicate that HDAC3 binds to regulatory regions associated with inflammatory, fibrotic, and steroid hormone signaling pathways and likely contributes to the modulation of transcriptional programs essential for uterine P4 responsiveness. Together, these findings support a link between HDAC3 chromatin occupancy and transcriptional programs associated with P4 responsiveness.

**FIGURE 4:**
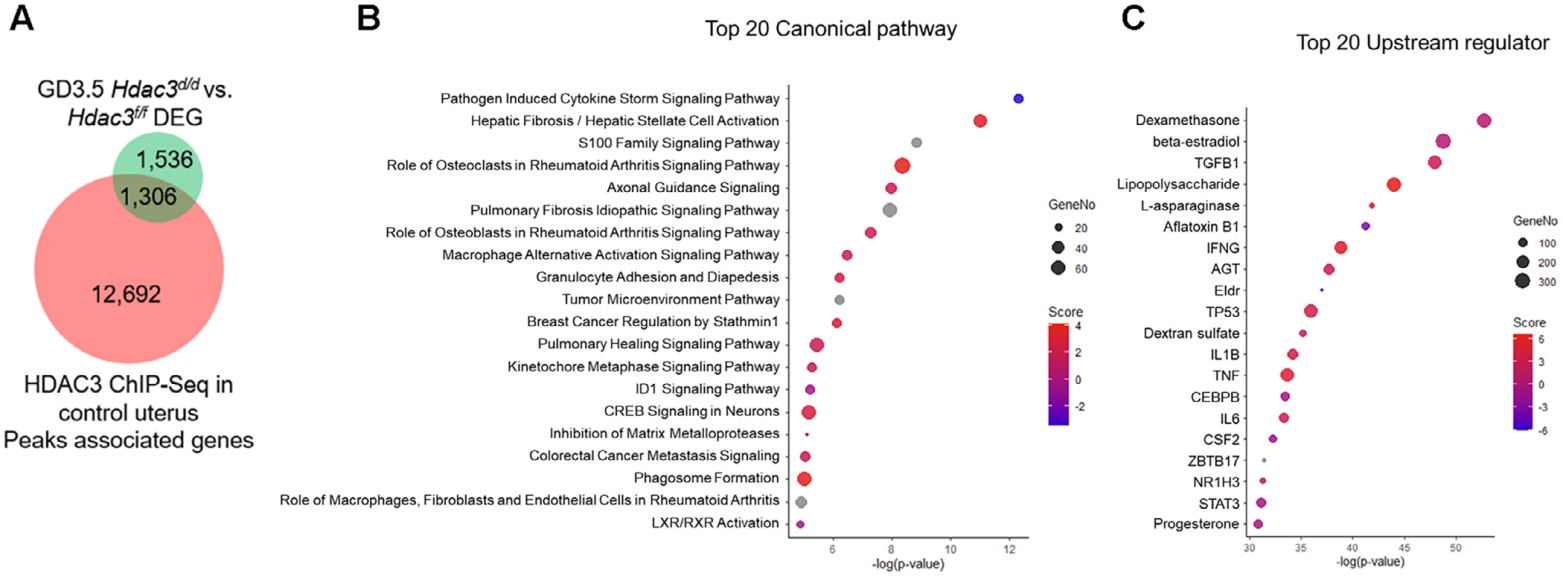
HDAC3 chromatin occupancy is associated with transcriptional programs regulating inflammation, fibrosis, and P4 signaling. (A) Venn diagram presenting the overlap between HDAC3 ChIP‐seq peak‐associated genes and DEGs identified in GD3.5 *Hdac3^f/f^* and *Hdac3^d/d^* uteri. (B and C) Dot plots showing the top 20 enriched canonical pathways (B) and predicted upstream regulators (C) among the 1306 DEGs associated with HDAC3 binding peaks. Pathway enrichment and upstream regulator analyses were performed using IPA. Dot color indicates the z‐score, and dot size reflects the number of genes associated with each pathway or regulator.

### 2.5 HDAC3 and PGR Co‐Occupy Chromatin and Cooperatively Regulate P4‐Responsive Genes in the Uterus

To determine whether HDAC3 and PGR coordinate transcriptional regulation through shared chromatin occupancy, we compared our HDAC3 ChIP‐seq dataset with published PGR ChIP‐seq data from the GD3.5 mouse uterus [26]. Strikingly, 75% (17,390 of 23,036) of HDAC3 binding peaks overlapped with PGR peaks, and 82% (17,390 of 21,327) PGR peaks overlapped with HDAC3 peaks, indicating extensive and coordinated chromatin binding between HDAC3 and PGR (Figure 5A). This extensive overlap supports the presence of a shared chromatin regulatory network. Using the Genomic Regions Enrichment of Annotations Tool (GREAT) [25], a total of 17,390 shared HDAC3‐PGR binding peaks were assigned to 12,990 predicted target genes. We then compared these 12,990 predicted target genes associated with HDAC3‐PGR co‐bound peaks with the 2842 DEGs identified by RNA‐seq analysis. Notably, 38% (1087 of 2842) of the DEGs resulting from uterine HDAC3 loss overlapped with the predicted HDAC3‐PGR co‐bound genes (Figure 5B), suggesting that a substantial subset of HDAC3‐dependent transcriptional changes are associated with HDAC3‐PGR chromatin interactions. Pathway and regulator analysis of HDAC3‐PGR co‐bound genes revealed enrichment of immune and fibrosis signaling pathways, β‐estradiol signaling, and suppression of P4 signaling, mirroring patterns observed among HDAC3‐regulated DEGs alone (Figure 5C,D). In addition, these co‐regulated genes exhibited downregulation of ERBB2 signaling and activation of retinoic acid receptor (RAR) signaling pathways (Figure 5C,D), both of which are known to influence endometrial differentiation and receptivity [27, 28]. A gene expression heatmap further illustrated differential regulation of genes associated with enhanced β‐estradiol signaling and suppression of P4 and STAT3 signaling in *Hdac3^d/d^* uteri (Figure 5E).

**FIGURE 5:**
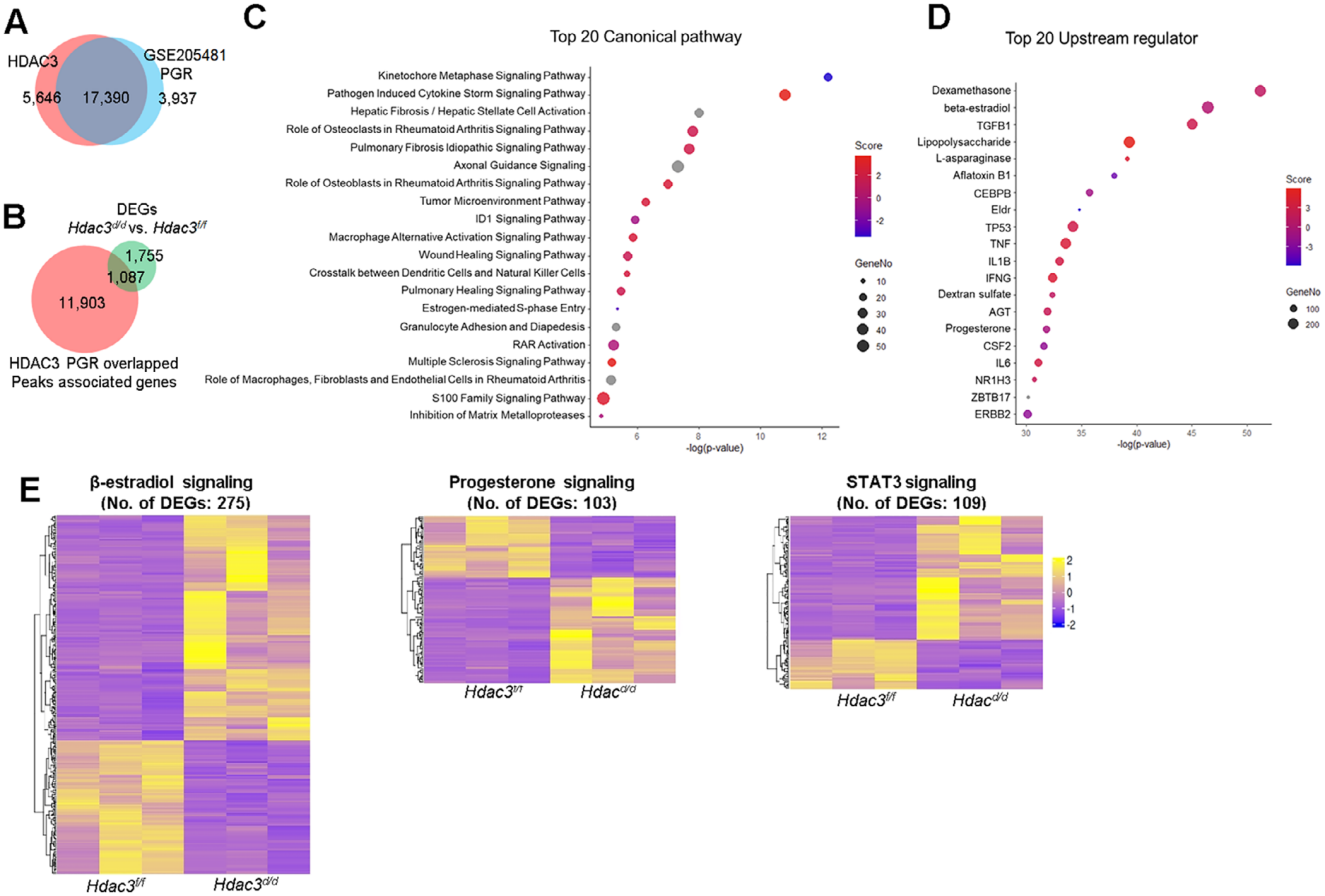
HDAC3‐PGR co‐occupancy is associated with transcriptional regulation of inflammation‐ and fibrosis‐related gene programs. (A) Venn diagram showing the overlap between HDAC3 and PGR ChIP‐seq binding peaks in the GD3.5 mouse uterus (data from GSE178542). (B) Venn diagram showing the overlap between the 12,990 predicted genes associated with shared HDAC3‐PGR binding peaks and the 2842 DEGs identified in *Hdac3^d/d^* uteri, yielding 1087 overlapping genes. (C) Dot plot depicting the top 20 canonical pathways enriched among the 1087 DEGs that overlap with HDAC3‐PGR peak‐associated genes. (D) Dot plot showing the top 20 predicted upstream regulators of these 1087 DEGs. Pathway enrichment and upstream regulator analyses were performed using Ingenuity Pathway Analysis (IPA). Dot color indicates the z‐score, and dot size reflects the number of genes associated with each pathway. (E) Heatmap showing differential expression of genes associated with β‐estradiol, progesterone, and STAT3 signaling.

Given the extensive overlap in HDAC3 and PGR chromatin occupancy, we hypothesized that HDAC3 may modulate PGR transcriptional activity by influencing its chromatin‐binding affinity. To test this hypothesis, we examined HDAC3 and PGR occupancy at four well‐known P4 target genes, *Fkbp5* [29, 30], *Lrp2* [31], *HOXA11* [32], and *Fst* [33, 34], in GD 3.5 *Hdac3^f/f^* and *Hdac3^d/d^* uteri using ChIP‐qPCR (Figure 6). At least one HDAC3‐PGR co‐bound region was identified at the promoter and/or gene body of each gene. Importantly, deletion of HDAC3 significantly reduced PGR chromatin binding at the examined regions of all four genes, with statistically significant decreases observed at the *Fkbp5* promoter (*Hdac3^f/f^*: 70.89 ± 32.78, *Hdac3^d/d^*: 13.35 ± 19.93, *p* < 0.05), *Fkbp5* gene body regions (*Hdac3^f/f^*: 41.65 ± 15.29, *Hdac3^d/d^*: 5.48 ± 8.36, *p* < 0.01), and *Lrp2* enhancer (*Hdac3^f/f^*: 5.49 ± 2.11, *Hdac3^d/d^*: 1.60 ± 1.47, *p* < 0.01). Together, these data link HDAC3 chromatin occupancy to the maintenance of PGR binding at key P4‐responsive loci and support a model in which HDAC3 contributes to sustaining PGR chromatin occupancy at key P4‐responsive genes in the uterus.

**FIGURE 6:**
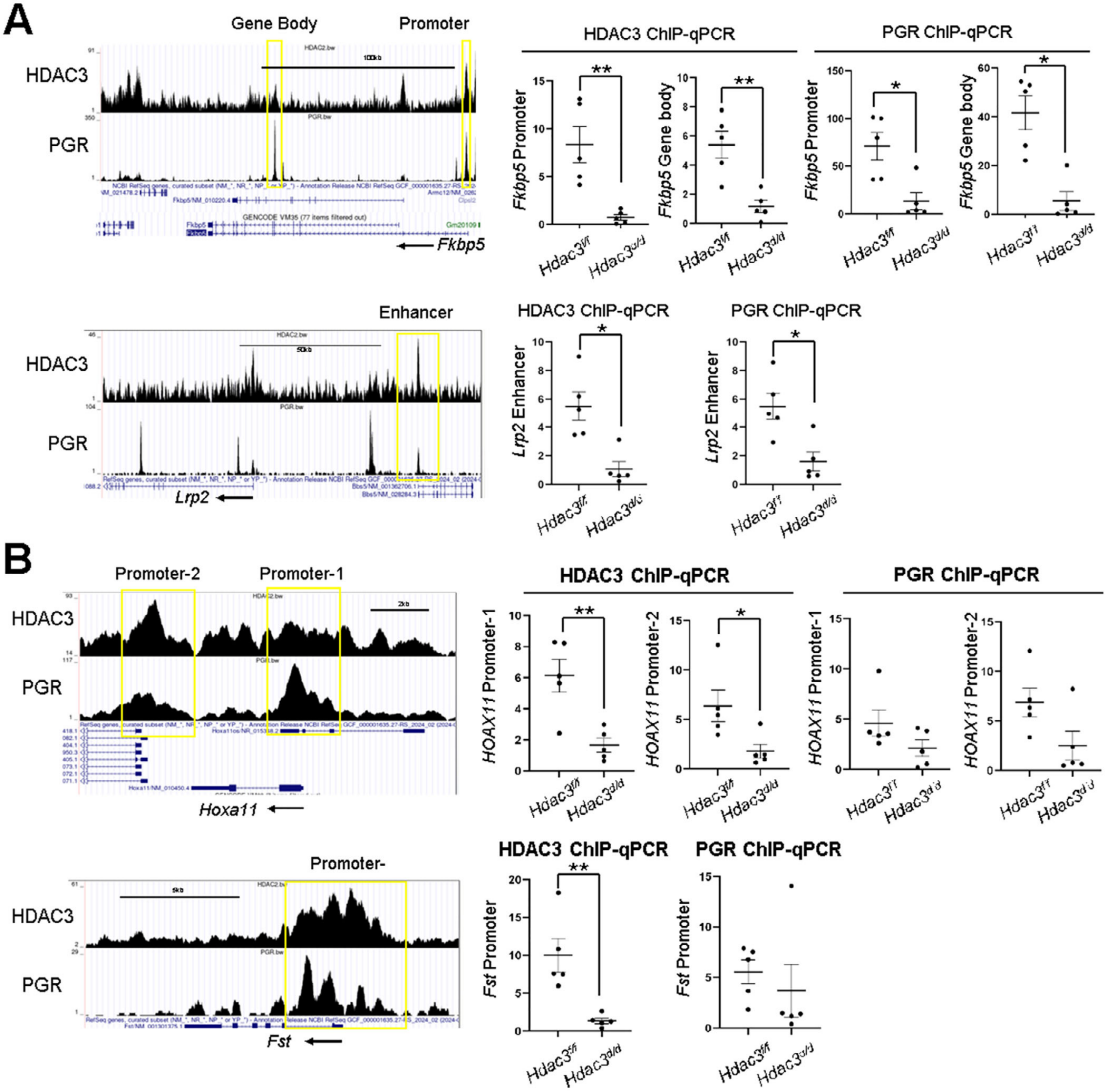
HDAC3 supports PGR chromatin occupancy at progesterone‐responsive genes. (A) ChIP‐qPCR validation of HDAC3 and PGR co‐occupancy at the promoter and/or gene body regions of four well‐characterized progesterone target genes in GD3.5 *Hdac3^f/f^* and GD3.5 *Hdac3^d/d^* mouse uteri. Upper panel shows ChIP‐qPCR analysis for *Fkbp5* promoter and lower panel shows analysis for *Lrp2* enhancer. (B) ChIP‐qPCR analysis of HDAC3 and PGR occupancy at HDAC3‐PGR co‐bound regions associated with *Hoxa11* and *Fst* in GD3.5 *Hdac3^f/f^* and *Hdac3^d/d^* uteri. Data are presented as mean ± SEM. * *p* < 0.05 and ** *p* < 0.01 by Student's *t*‐test.

### 2.6 HDAC3 Associates With PGR and Reciprocally Regulates Their Uterine Expression

Having established extensive chromatin co‐occupancy between HDAC3 and PGR and shown that loss of HDAC3 attenuates PGR chromatin binding, we next investigated whether HDAC3 associates with PGR. To this end, we coexpressed HDAC3 with either PGR‐A or PGR‐B isoforms in HEK293FT cells and performed co‐immunoprecipitation analysis. HDAC3 preferentially associated with the PGR‐A isoform, as reciprocal immunoprecipitation of either protein efficiently pulled down the other, indicating formation of a protein complex (Figure 7A).

**FIGURE 7:**
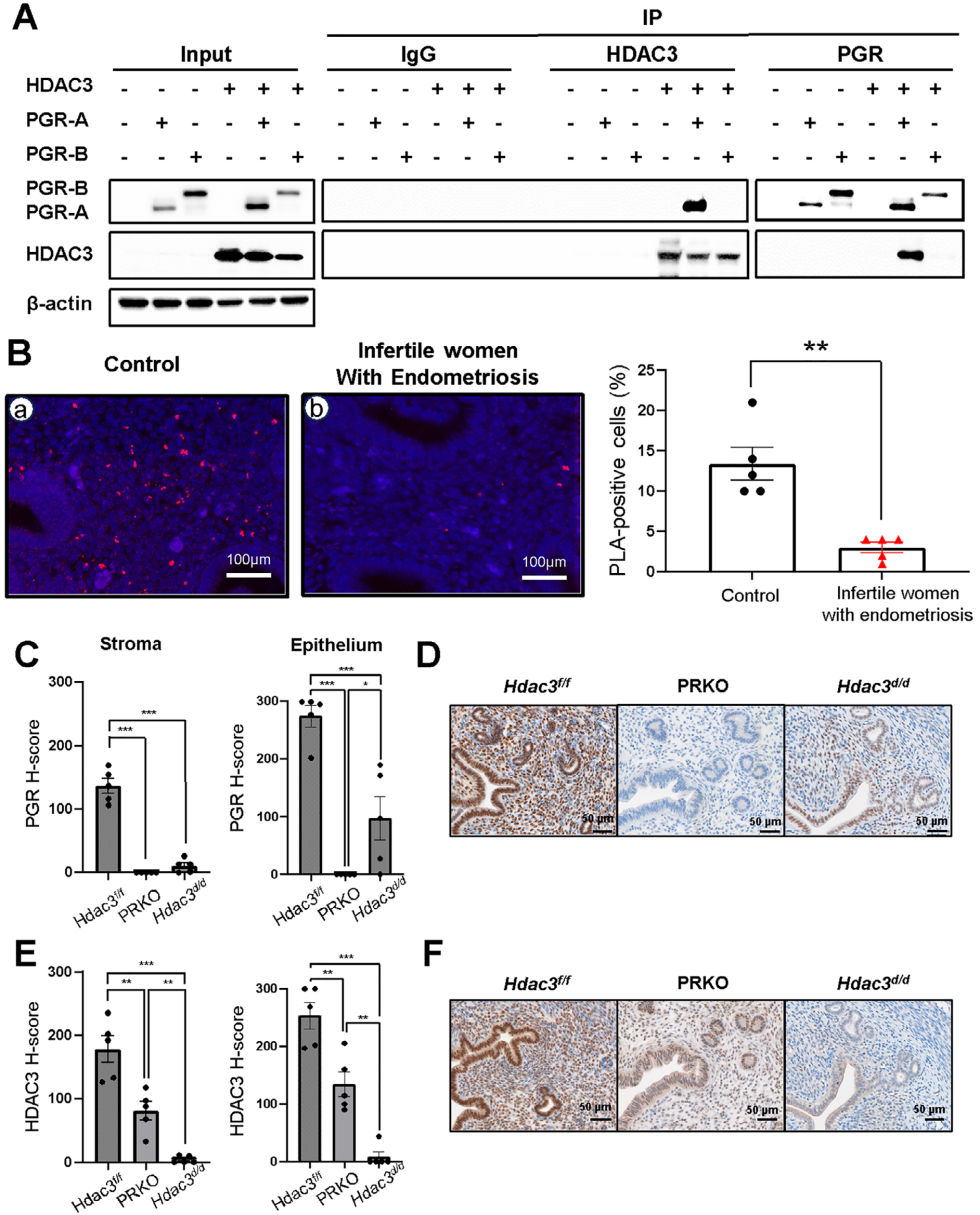
HDAC3 associates with PGR and reciprocally regulates their uterine expression. (A) Co‐immunoprecipitation (Co‐IP) assays demonstrating preferential association of HDAC3 with the PGR‐A isoform in HEK293FT cells co‐transfected with HDAC3 and PGR‐A and PGR‐B expression plasmids. (B) Proximity ligation assay (PLA) of PGR and HDAC3 in human mid‐secretory endometrium from controls (*n* = 5) and infertile women with endometriosis (*n* = 5), along with quantification of the resulting PLA signals. ** *p* < 0.01 by Student's *t*‐test. (C) Semi‐quantitative analysis of stromal (left) and epithelial (middle) PGR expression in *Hdac3^f/f^*, PRKO and *Hdac3^d/d^* mouse uteri at GD3.5 (*n* = 5 per group). (D) Representative IHC images illustrating the expression of PGR in *Hdac3^f/f^*, PRKO, and *Hdac3^d/d^* mouse uteri. (E) Semi‐quantitative analysis of stromal (left) and epithelial (middle) HDAC3 expression in *Hdac3^f/f^*, PRKO, and *Hdac3^d/d^* mouse uteri at GD3.5 (*n* = 5 per group). (F) Representative IHC images demonstrating HDAC3 expression in *Hdac3^f/f^*, PRKO, and *Hdac3^d/d^* mouse uteri. Data are presented as mean ± SEM. * p < 0.05, ** p < 0.01, *** p < 0.001. PLA data were analyzed using Student's *t*‐test, and IHC data were analyzed using one‐way ANOVA followed by Tukey's post hoc test.

To assess this association in a physiologically relevant context, we performed an in situ proximity ligation assay (PLA) using the Duolink kit [35], which enables detection of protein–protein interactions at single‐molecule resolution within intact endometrial compartments. PLA analysis revealed robust HDAC3‐PGR proximity signals in the stromal compartment of mid‐secretory phase endometrium from control women (Figure 7B), consistent with the known role of stromal PGR signaling in endometrial receptivity. In contrast, these stromal signals were markedly reduced in infertile women with endometriosis (3.00 ± 0.63%) compared to controls (13.40 ± 2.04%, *p* = 0.0012) (Figure 7B). Notably, PLA signals were predominantly localized to stromal nuclei, supporting compartment‐specific association of HDAC3 and PGR in cells critical for decidualization. These findings support an association between HDAC3 and PGR in the human endometrium and demonstrate that this interaction is significantly disrupted in endometriosis, supporting a functional role for the HDAC3‐PGR complex in maintaining P4 responsiveness.

These findings raised the possibility that HDAC3 and PGR may be mutually required to maintain stable expression in vivo, consistent with their coordinated expression patterns observed in human and mouse endometrium. *Pgr* knockout (PRKO) mice exhibit pleiotropic defects in the female reproductive tract, including complete loss of uterine P4 responses [36, 37]. To examine whether HDAC3 and PGR regulate each other's uterine expression, we performed IHC with H‐score quantification in uteri from *Hdac3^f/f^*, PRKO, and *Hdac3^d/d^* mice at GD3.5. In *Hdac3^f/f^* uteri, PGR expression was robust in both the stromal (136.14 ± 12.99) and epithelial compartments (274 ± 20.66). As expected, PGR expression was completely absent in both stroma (0.00 ± 0.00, *p* < 0.001) and epithelium of PRKO mice. Interestingly, PGR expression was significantly reduced in the stroma (10.50 ± 5.24, *p* < 0.001) and epithelium (97.13 ± 42.14, *p*< 0.001) of *Hdac3^d/d^* mice compared to *Hdac3^f/f^* (Figure 7C,D). Conversely, HDAC3 expression was high in *Hdac3^f/f^* uteri (stromal: 177.82 ± 23.45; epithelium: 253.63 ± 25.50), nearly absent in *Hdac3^d/d^* mice (stroma: 4.34 ± 2.97; epithelium: 8.68 ± 9.71; both *p* < 0.001), and significantly reduced in PRKO mice (stroma: 80.83 ± 16.12; epithelium: 134.00 ± 24.09; both *p* < 0.01) compared to *Hdac3^f/f^* (Figure 7E,F). Together, these data support a reciprocal regulatory relationship between HDAC3 and PGR important for uterine P4 responsiveness.

## 3 Discussion

Our findings identify HDAC3 as a coregulator of PGR signaling that influences PGR protein abundance and chromatin occupancy, providing a mechanistic link between HDAC3 dysregulation and P4 resistance in the uterus (Figure 8). Although the precise mechanism remains to be defined, several observations suggest that HDAC3 regulation of PGR extends beyond its canonical histone deacetylase activity. Decidualization is accompanied by increased histone acetylation [38], and HDAC inhibitors can promote decidualization of human endometrial stromal cells in vitro [39]. Moreover, HDAC3 shuttles between the nucleus and cytoplasm and can regulate non‐histone proteins [40], with deacetylase‐independent transcriptional functions mediated through interactions with transcription factors [41]. Together with our demonstration of HDAC3‐PGR association and chromatin co‐occupancy, these findings support a broader coregulatory role for HDAC3 in uterine PGR function.

**FIGURE 8:**
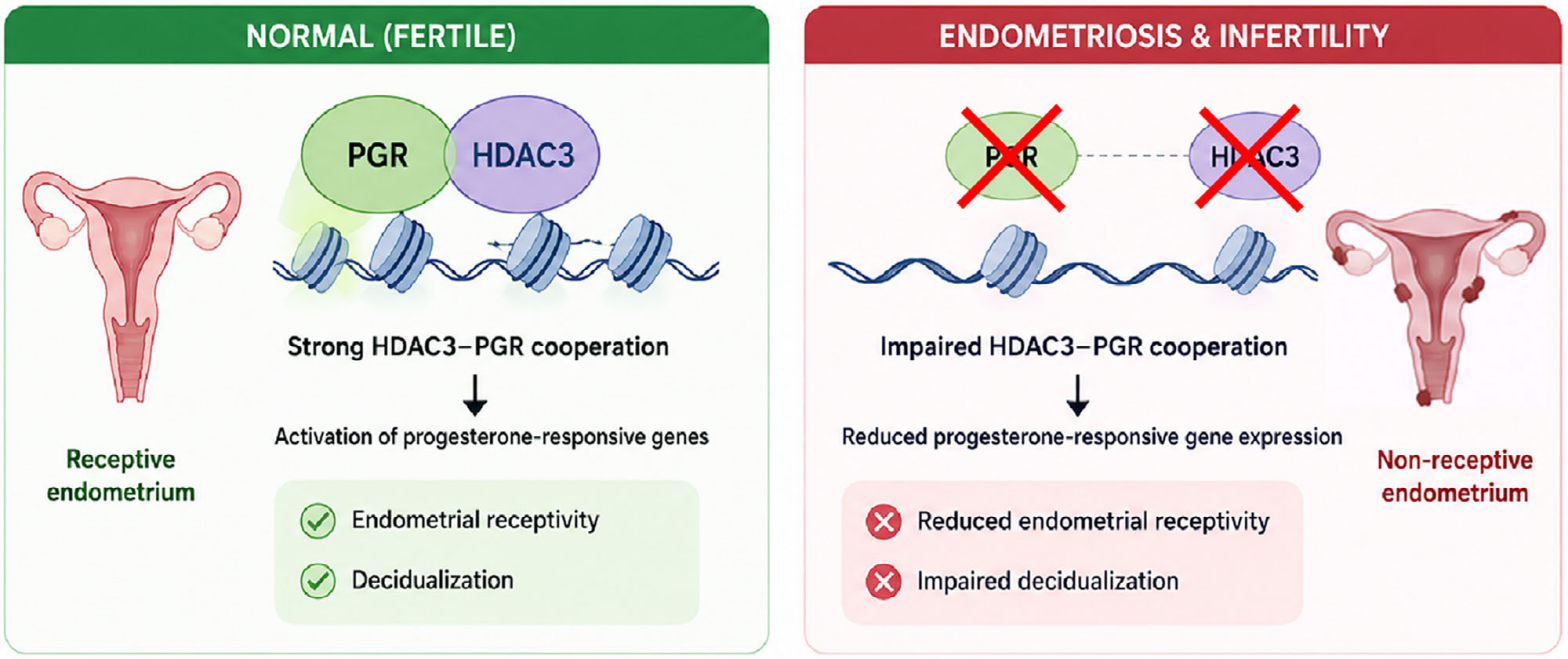
Proposed model of HDAC3‐PGR cooperation in regulating uterine P4 responsiveness and endometrial receptivity. In the receptive endometrium, HDAC3 associates with PGR and co‐occupies regulatory regions of P4‐responsive genes, supporting PGR chromatin occupancy and transcriptional programs required for normal P4 responsiveness and endometrial receptivity. In infertile women with endometriosis, reduced stromal HDAC3 and PGR expression and decreased HDAC3‐PGR association are associated with impaired P4 signaling. Loss of uterine HDAC3 reduces PGR expression and chromatin occupancy and is accompanied by dysregulation of P4‐responsive transcriptional programs, including increased inflammatory and fibrotic signaling. Together, these findings support a model in which disruption of HDAC3‐PGR cooperation contributes to P4 resistance and impaired endometrial receptivity associated with endometriosis‐related infertility. ChatGPT (OpenAI) was used to assist in generating the initial version of this schematic, which was subsequently modified and finalized by the authors.

Epigenetic remodeling is essential for endometrial receptivity and decidualization [38, 39, 42, 43]. HDACs regulate chromatin structure and transcriptional programs involved in cell proliferation and differentiation [44, 45], and dysregulated HDAC activity has been implicated in endometrial disorders, including endometrial cancer, endometriosis, and infertility [8, 17, 46, 47]. Our previous studies demonstrated that uterine‐specific loss of HDAC3 impairs endometrial receptivity and decidualization and causes infertility [8]. The present findings extend these observations by linking HDAC3 deficiency to reduced PGR expression and occupancy, altered P4‐responsive transcription, and activation of inflammatory and fibrotic pathways. Thus, HDAC3 appears to integrate chromatin regulation with PGR‐dependent transcriptional programs required for uterine receptivity.

Among mammalian HDACs, HDAC3 is reduced in the eutopic endometrium of infertile women with endometriosis [8], whereas HDAC1 and SIRT1 are increased [17, 47, 48]. The mechanisms responsible for selective HDAC3 downregulation remain unclear. HDAC3 stability and activity depend on interactions with NCoR/SMRT corepressor complexes and are also regulated by post‐translational modifications [49, 50]. In the present study, HDAC3 and PGR protein levels were positively correlated in human endometrium, and reciprocal reductions were observed in *Hdac3^d/d^* and PRKO uteri. These findings suggest coordinated regulation of HDAC3 and PGR expression and raise the possibility that disruption of this relationship contributes to impaired P4 responsiveness.

Epithelial PGR expression was elevated in a subset of infertile women with endometriosis, consistent with prior reports of aberrant PGR regulation rather than uniform receptor loss. However, Group 1 in Figure 1 exhibited low epithelial PGR expression comparable to controls but showed no significant correlation between HDAC3 and PGR because HDAC3 expression was variable. Cases with relatively high HDAC3 and low PGR may represent a more physiologically preserved epithelial state, as low epithelial PGR expression is characteristic of the mid‐secretory endometrium. In contrast, cases with low HDAC3 and low PGR may represent a distinct form of epithelial dysfunction in which reduced HDAC3 compromises downstream transcriptional competence despite an epithelial PGR expression pattern that remains comparable to controls. Thus, P4 resistance in these cases could potentially arise from impaired coregulator function rather than abnormal PGR abundance itself. However, because Group 1 retains an overall epithelial PGR expression pattern similar to that of controls and substantial variability in HDAC3 expression, we did not focus on this subgroup in the present study. Future studies with larger cohorts and functional analyses will be required to determine whether reduced HDAC3 identifies a distinct molecular subtype within the PGR‐low epithelial group. More broadly, the distinct HDAC3‐PGR correlation patterns observed between stromal and epithelial compartments may reflect cell type‐specific regulation of P4 signaling. Our single‐cell analysis demonstrated consistently strong positive correlations between HDAC3 and PGR expression in stromal cells from both controls and infertile women with endometriosis, suggesting that coordinated HDAC3‐PGR regulation is a relatively stable feature of stromal P4 signaling. This relationship is particularly relevant because stromal PGR signaling plays a central role in decidualization and endometrial receptivity. In contrast, the relationship between HDAC3 and PGR was more heterogeneous in epithelial cells. Because epithelial PGR expression is dynamically regulated and normally reduced during the mid‐secretory phase, its expression and activity may be influenced by additional cell type–specific regulatory mechanisms beyond HDAC3 expression alone. These may include receptor turnover, coregulator availability, chromatin regulation, and stromal‐epithelial paracrine signaling. Thus, the abnormal epithelial PGR expression and strengthened HDAC3‐PGR correlation observed in a subset of infertile women with endometriosis may reflect altered epithelial P4 signaling rather than simply changes in PGR abundance. Further cell type–specific studies will be required to define the mechanisms underlying differential HDAC3‐PGR regulation in stromal and epithelial compartments.

This observation suggests that P4 resistance may arise from defective coregulator function and chromatin engagement rather than reduced PGR abundance alone. Based on these observations, we propose that altered steroid hormone receptor signaling characteristic of endometriosis [2] may converge to reduce HDAC3 abundance and/or complex integrity, thereby weakening PGR function and promoting P4 resistance. Importantly, the identification of HDAC3 as a regulator of PGR function suggests that modulation of this axis may represent a potential therapeutic strategy. Given the reversible nature of chromatin‐associated regulatory mechanisms, therapeutic strategies that restore appropriate HDAC3 function or HDAC3‐PGR cooperation warrant investigation as potential approaches to enhance P4 responsiveness in endometriosis.

Several limitations of this study should be acknowledged. Human sample analyses were limited in size and restricted to the mid‐secretory phase, which may not capture the full spectrum of hormonal variability or disease heterogeneity. Co‐immunoprecipitation experiments were performed in HEK293FT cells rather than primary uterine cells and therefore may not fully recapitulate physiological protein–protein interactions in the endometrium. Additionally, although we identified extensive chromatin co‐occupancy of HDAC3 and PGR, we did not directly assess histone or non‐histone post‐translational modifications such as acetylation or lactylation that could influence PGR activity.

Future studies employing cell type–specific and temporally controlled genetic models will be essential to dissect stromal versus epithelial roles of HDAC3 in regulating PGR signaling during implantation. High‐resolution epigenomic approaches, including CUT&RUN or single‐cell multi‐omics in human and mouse endometrium, will further clarify how HDAC3‐PGR complexes shape chromatin accessibility and transcriptional networks. Investigating potential non‐histone regulatory functions of HDAC3, including possible delactylase activity and modulation of PGR post‐translational modifications, may reveal how metabolic cues such as hypoxia‐induced lactate accumulation integrate with hormonal signaling. Finally, pharmacologic or genetic strategies aimed at restoring HDAC3 function in endometriosis models may determine whether targeting the HDAC3‐PGR axis can reverse P4 resistance and improve endometrial receptivity, thereby informing translational approaches for infertility associated with endometriosis.

In summary, this study identifies HDAC3 as a critical transcriptional coregulator of PGR signaling in the uterus. Through association and coordinated chromatin binding, HDAC3 contributes to maintaining appropriate PGR protein levels and genomic occupancy required for endometrial receptivity. Loss of HDAC3 disrupts these processes, leading to aberrant activation of inflammatory and fibrotic pathways characteristic of P4 resistance in both human and mouse uteri. Reduced HDAC3 expression in the eutopic endometrium of infertile women with endometriosis, together with reciprocal downregulation of PGR, underscores the importance of this regulatory axis in uterine function. Together, these findings define HDAC3‐PGR cooperation as an important regulatory mechanism underlying P4 responsiveness and provide a framework for exploring this pathway as a potential therapeutic target for endometrial dysfunction in endometriosis.

## 4 Materials and Methods

### 4.1 Human Endometrial Tissues

Twenty‐two control women and 43 infertile women with endometriosis were recruited at Atrium Health Wake Forest Baptist (Winston‐Salem, NC), where the study was reviewed and approved by the Institutional Review Boards. Written informed consent was obtained from all human subjects prior to tissue acquisition. The presence or absence of disease was confirmed in all endometriosis cases during surgery. The control group included women who were laparoscopically negative for disease. Women using an intrauterine device (IUD) or hormonal therapies in 3 months preceding surgery were excluded from this study. Histologic dating of endometrial samples was performed by an experienced fertility specialist (B.A.L.). Endometrial biopsies taken during secretory phase were fixed in 4% paraformaldehyde, dehydrated and embedded in wax for IHC and multiplex staining.

### 4.2 Mice

All mice were maintained in the animal facility of the Institutional Animal Care and Use Committee of University of Missouri. Our study exclusively examined female mice because the disease modeled is only relevant in females. *Hdac3^f/f^* mice were generously shared by Dr. Eric Olson at UT Southwestern Medical Center. *Pgr^cre/+^* mice [51] were crossed with *Hdac3^f/f^* mice [8] to generate uterine‐specific *Hdac3* knockout mice, *Pgr^cre/+^ Hdac3^f/f^* (*Hdac3^d/d^*). The *Hdac3^f/f^* female littermates were used as control mice. Because insertion of Cre recombinase into the first exon disrupts endogenous *Pgr* expression [51], we used *Pgr^cre/cre^* females as PRKO mice. Mice were bred on a mixed background of the mouse strains C57Bl/6 and SV129 under controlled humidity and temperature conditions.

### 4.3 Mouse Tissue Collection

Eight‐week‐old wild‐type (WT) control*, Hdac3^d/d^*, and PRKO female mice were mated with WT males. The copulation plug was checked daily. The morning when the plug was observed was defined as GD 0.5. The mouse uteri were collected at GD 3.5. The collected uteri were either fixed in 4% (w/v) paraformaldehyde (PFA; Thermo Fisher Scientific, Waltham, MA) for IHC or stored at −80°C for molecular analyses.

### 4.4 Immunohistochemical Analysis

Paraffin‐embedded mouse endometrial tissues were sectioned at 5 µm, deparaffinized, rehydrated, and blocked with 10% normal goat serum in PBS for 2 h at room temperature. Sections were incubated with anti‐HDAC3 (1:500; Proteintech, 10255‐1‐AP) or anti‐PGR (1:500; Cell Signaling Technology, 8757) antibodies overnight, followed by biotinylated anti‐rabbit IgG secondary antibody (1:500; Vector Laboratories, BA‐9200) and HRP‐streptavidin (1:1000; Invitrogen, 43–4323). Signals were developed using the Vectastain Elite DAB kit (Vector Laboratories, SK‐4100). Sections were counterstained with hematoxylin, dehydrated, and coverslipped.

### 4.5 Multiplex Immunofluorescence

Multiplex immunofluorescence was performed using the Opal 7‐Color IHC Kit (Akoya Biosciences, NEL811001KT) according to the manufacturer's instructions. Tissue sections were deparaffinized, rehydrated, fixed in 10% neutral buffered formalin, and subjected to antigen retrieval in AR6 buffer (Akoya Biosciences) at 95°C for 15 min. Sections were blocked with Antibody Diluent/Block solution and sequentially incubated with antibodies against CD10 (1:100; Cell Signaling Technology, 65534), pan‐keratin (1:100; Cell Signaling Technology, 4545), PGR (1:200; Cell Signaling Technology, 8757), and HDAC3 (1:100; Cell Signaling Technology, 85057). Signals were detected using Opal Anti‐MS + Rb HRP and Opal 480, 520, 570, and 620 fluorophores, respectively (Akoya Biosciences). After final antigen retrieval, sections were counterstained with Spectral DAPI, mounted with ProLong Diamond Antifade Mountant (Invitrogen, P36970), and scanned using the PhenoImager HT 2.0 (Akoya Biosciences).

### 4.6 Duolink in Situ PLA Analysis

Duolink in situ PLA was performed according to the manufacturer's instructions (Sigma‐Aldrich, Watertown, MA) as previously described [35]. Briefly, paraformaldehyde‐fixed tissues were blocked with blocking solution. Primary antibodies, against PGR and HDAC3, were applied, and the tissues were incubated with PLUS and MINUS secondary PLA probes against rabbit and mouse IgGs. The incubation was followed by hybridization and ligation, and then amplification was performed. After mounting with Duolink mounting medium, samples were scanned with the Phenolmager HT 2.0 (Akoya Biosciences).

### 4.7 Quantification Analysis of IHC, PLA, and Multiplex by Digital Image Analysis Software

IHC, PLA, and multiplex immunofluorescence images were quantified using Visiopharm software (Visiopharm Integrator System, Hoersholm, Denmark). Epithelial and stromal regions of interest (ROIs) were manually defined, and nuclei were identified using the deep‐learning‐based Nuclear Detection AI algorithm (#10169). For IHC, more than 300 epithelial and 1500 stromal cells were analyzed per sample. DAB‐positive cells were classified as weak (+1), moderate (+2), or strong (+3), and H‐scores were calculated as: (1 × % weak) + (2 × % moderate) + (3 × % strong), yielding scores from 0 to 300. PLA signals were quantified within the corresponding ROIs.

For multiplex immunofluorescence, epithelial and stromal cells were segmented and fluorescence intensities for Opal 480, 520, 570, and 620 were quantified. More than 1000 epithelial and 1500 stromal cells were analyzed per sample after exclusion of artifacts. Correlations between PGR (Opal 520) and HDAC3 (Opal 620) fluorescence intensities were assessed at the single‐cell level. t‐SNE analysis based on PGR and HDAC3 intensities was performed using the Visiopharm QC tool.

### 4.8 Construction and Sequencing of Directional mRNA‐seq Libraries

RNA‐seq libraries were prepared from 500 ng total RNA using the KAPA mRNA HyperPrep Kit (Kapa Biosystems). RNA was fragmented to 300–400 bp, and cDNA was ligated to IDT for Illumina TruSeq UD Indexed adapters. Library quality and quantity were assessed using an Agilent DNA High Sensitivity chip and QuantiFluor dsDNA System (Promega). Indexed libraries were pooled and sequenced as 50‐bp paired‐end reads on an Illumina NovaSeq 6000 to an average depth of 50 million reads per sample. Reads were demultiplexed and converted to FASTQ format using Bcl2fastq v1.9.0.

### 4.9 RNA‐Seq Analysis

The total RNA was isolated from *Hdac3^f/f^* and *Hdac3^d/d^* uteri at GD3.5 using the RNeasy Total RNA Isolation Kit (Qiagen, Germany). Low‐quality reads (average quality score < 20) were removed, and reads were aligned to the mm39 genome using STAR v2.7.9a [52]. DEGs were identified using edgeR [53] with max counts ≥ 50, fold change of > 2 or < ‐2, FDR < 0.05. PCA and heatmap were generated using edgeR and ComplexHeatmap package [54], respectively. The significantly enriched pathways and upstream regulators based on the DEGs were identified using Ingenuity Pathway Analysis (IPA, Qiagen, Germany) and dot plots were generated using Tidyverse [55].

### 4.10 RT‐qPCR

Note that 1 µg of total RNA was reverse transcribed to cDNA using M‐MLV reverse transcriptase kit (Thermo Fisher Scientific, Waltham, MA, 28025013) according to the manufacturer's instructions. Quantitative real‐time PCR was performed using the Applied Biosystems SYBR Green Universal Master Mix (Fisher Scientific, Waltham, MA, 43‐091‐55) and specific primers to examine the relative expression of target genes. Experimental gene expression data were normalized against the constitutively expressed ribosomal gene *Rpl7*.

### 4.11 Chromatin Immunoprecipitation and Sequencing (ChIP‐Seq) and ChIP‐qPCR

The whole uterine tissues of GD3.5 *Hdac3^f/f^* and *Hdac3^d/d^* mice were processed for ChIP analysis using ChIP‐IT High Sensitivity (Active Motif, Carlsbad, CA 53040) following the vendor's manual. Briefly, uterine tissues were freshly fixed with 1% PFA (w/v) at room temperature (RT) for 15 min. Chromatin was extracted by homogenization and fragmented by sonication (10 cycles at 50% amplitude). For ChIP‐qPCR, 10 µg of chromatin per sample from *Hdac3^f/f^* or *Hdac3^d/d^* uterus was used (*n* = 5 samples per group). For ChIP‐Seq, 30 µg of chromatin from control, *Hdac3^f/f^*, mice was used (*n* = 2 samples). Note that 0.5 µg PGR antibody (Cell Signaling Technology, Danvers, MA, CS8757) or 1 µg HDAC3 (Cell Signaling Technology, CS85057) was added for one ChIP sample; 5% of the sonicated chromatin was directly purified as input chromatin. The antibody‐immunoprecipitated chromatin was enriched and purified for further analysis.

Two HDAC3 ChIP and one input of *Hdac3^f/f^* GD3.5 mouse uteri were submitted for ChIP‐sequencing. Library construction and sequencing was performed by the Genomics Technology Core at the University of Missouri. Libraries were constructed following the manufacturer's protocol with reagents supplied in the NEBNext Ultra II DNA Library Prep Kit for Illumina (New England Biolabs, New England Biolabs, Ipswich, MA, E7103). Briefly, DNA fragments were end repaired/dA‐tailed followed by adaptor ligation. The final amplified cDNA constructs were purified by addition of AxyPrep Mag PCR Clean‐up beads, selecting for an insert size of ∼250 bp. Libraries were quantified with the Qubit High Sensitivity (HS) DNA Kit (Invitrogen, Waltham, MA) and the fragment size was analyzed on a 5,200 Fragment Analyzer (Agilent, Santa Clara, CA). Libraries were diluted according to the standard protocol for sequencing on the NovaSeq 6000 (Illumina, San Diego, CA).

### 4.12 ChIP‐seq Analysis

The HDAC3 ChIP and input sequencing reads were filtered by removing low quality reads (average quality score < 20) and aligned to the mm39 mouse genome and assembled using bowtie [56]. The duplicate reads were removed using Picard tools (Broad Institute, https://broadinstitute.github.io/picard/faq.html). The uniquely mapped reads were extended to 300 bp by SAMtools [57]. The peaks were called by MACS2 [58] with FDR cutoff at 0.001 and input as control. The genome distribution of the peaks and pathway analysis of peak associated genes were calculated by ChIPseeker [59].

PGR ChIP‐Seq raw reads in GD3.5 mouse uterus were downloaded from GEO accession GSE178542 [26], and processed similarly for peak calling. The overlapped peaks between PGR and HDAC3 were defined as regions with a minimum overlap of 1 bp. The peak associated genes were annotated based on their direct overlap with gene regulatory domains using GREAT (Genomic Regions Enrichment of Annotations Tool) with the basal plus extension setting [25]. Venn diagrams were generated using BioVenn [60] to compare genes associated with HDAC3 ChIP‐Seq peaks in control uterus samples to DEGs between wild‐type (GD3.5 *Hdac3^f/f^*) and the knock‐out (*Hdac3^d/d^*) data sets. The overlapping genes underwent core expression analysis in IPA to identify relevant canonical pathways and upstream regulators.

### 4.13 ChIP‐qPCR Analysis

The PGR or HDAC3 antibody targeted chromatin, and input chromatin from *Hdac3^f/f^* and *Hdac3^d/d^* mice (*n* = 5 per group) was applied for RT‐qPCR using SsoAdvanced Universal SYBR Green Supermix (Bio‐Rad, Hercules, CA). SYBR Green primers targeting PGR and HDAC3 co‐binding peaks near genes of interest were designed using SnapGene Viewer 6.0 and synthesized by Sigma‐Aldrich. Relative enrichment was calculated using the ΔΔCt method, with the Mouse Negative Control Primer Set 1 (Active Motif, Carlsbad, CA, 71011) as a negative reference. The resulting values represent the fold enrichment of antibody‐bound chromatin over input chromatin.

### 4.14 Cell Culture and Transfection

HEK293FT cells were cultured in Dulbecco's modified Eagle's medium/nutrient Mixture F‐12 (Gibco BRL, Gaithersburg, DMEM/F‐12) with 10% (v/v) fetal bovine serum (FBS; Gibco BRL, Gaithersburg, MD), and 1% (v/v) penicillin‐streptomycin (P/S; Gibco BRL, Gaithersburg, MD) at 37°C under 5% CO_2_. After 4 h of starvation in basal DMEM/F‐12 media, HEK293FT cells cultured in six‐well plates were transfected with 1 µg of PGR‐A, 3 µg of PGR‐B, 3 µg of HDAC3, PGR‐A with HDAC3, and PGR‐B with HDAC3 using Lipofectamine 2000 reagent (Invitrogen Corp. Carlsbad, CA) in accordance with the manufacturer's instructions.

### 4.15 Co‐Immunoprecipitation (Co‐IP) Analysis

Co‐IP was performed as described previously [48]. Cell lysates (1 mg) were incubated overnight at 4°C with anti‐HDAC3 or anti‐PGR antibody (1 µg) and Protein A Plus Agarose (Thermo Scientific). Immunoprecipitated proteins were separated by SDS‐PAGE, transferred to PVDF membranes, and immunoblotted with antibodies against PGR (1:1000; Cell Signaling Technology, 8757) or HDAC3 (1:1000; Cell Signaling Technology, 85057), followed by HRP‐conjugated secondary antibody (1:5000; Thermo Fisher Scientific, 31460). Signals were detected using Amersham ECL Prime reagent (Cytiva) and imaged with a ChemiDoc Touch Imaging System (Bio‐Rad).

### 4.16 Statistical Analysis

Two‐group comparisons were performed using unpaired Student's *t*‐test with unequal variance, and multiple‐group comparisons were performed using one‐way ANOVA followed by Tukey's post hoc test. Correlations were assessed using Pearson's correlation coefficient. Data are presented as mean ± SEM, with *p* < 0.05 considered statistically significant. Analyses were performed using GraphPad Prism v10.2.0.

## Author Contributions

Jung‐Yoon Yoo and Jae‐Wook Jeong were responsible for the concept of the study. Steven L. Young and Bruce A. Lessey collected human samples. Sohyeon Yun, Rong Li, Soo Lim Kim, Loan Thi Kim Nguyen, Shamsun Nahar, Keun Cheon Kim, DaHye Kang, Eunhee M. Jeong, and Tae Hoon Kim carried out experiments. Sohyeon Yun, Rong Li, Soo Lim Kim, Loan Thi Kim Nguyen, Shamsun Nahar, and Keun Cheon Kim analyzed data. Sohyeon Yun, Rong Li, Soo Lim Kim, Lisa Brennaman, Mistie P. Mills, Tae Hoon Kim, Jung‐Yoon Yoo, and Jae‐Wook Jeong prepared the manuscript. All authors have read and approved the final manuscript.

## Funding

This work was supported by the Eunice Kennedy Shriver National Institute of Child Health and Human Development (NICHD) (R01HD102170, R01HD101243, P01HD106485, and R01HD108895 to J.‐W.J.) and the National Research Foundation of Korea (NRF) grant funded by the Korean government (MSIT) (No. 2022R1A2C1091055 to J.‐Y.Y.). The content is solely the responsibility of the authors and does not necessarily represent the official views of the National Institutes of Health.

## Ethics Statement

The study was reviewed and approved by the Institutional Review Boards at Atrium Health Wake Forest Baptist (IRB00057549). Informed consent was obtained from all participants in the study. All animal experimental procedures were approved by the Institutional Animal Care and Use Committee (Protocol 65323) of University of Missouri.

## Conflicts of Interest

The authors declare no conflicts of interest.

## Data Availability

The datasets generated and analyzed during the current study, including IHC results from human endometrial tissue, are available from the corresponding author upon reasonable request. These data are stored in compliance with institutional policies regarding data sharing and confidentiality. The raw data underlying the findings of this study are not publicly available due to restrictions based on patient privacy and institutional policies but can be shared in anonymized form upon request. The raw and processed RNA‐Seq (GSE308870) and ChIP‐Seq (GSE308869) data have been deposited in the Gene Expression Omnibus. Values for all data points in graphs are reported in the Supporting Data Values file.
